# Minimally Invasive Valve-Sparing Approach for Mitral Leaflet Perforation

**DOI:** 10.1016/j.jaccas.2025.105585

**Published:** 2025-10-29

**Authors:** Alexandra Murillo-Solera, Oscar Holmvard, Ahmet Bilgili, Vaishnavi Karanam, Juan Carlos Tafur-Mejia, Thomas M. Beaver

**Affiliations:** Division of Cardiovascular Surgery, Department of Surgery, University of Florida, Gainesville, Florida, USA

**Keywords:** imaging, mitral valve, valve repair

## Abstract

**Objective:**

To describe the case of a 52-year-old man who developed severe mitral regurgitation 4 years after undergoing aortic valve replacement, maze procedure, and mitral vegetation removal during surgery for infective endocarditis; the mitral regurgitation was due to a perforation of the anterior mitral leaflet (A2) identified on transthoracic echocardiography.

**Key Steps:**

Key procedural steps included: 1) right minithoracotomy access; 2) adhesiolysis and wedge resection of pleural perforations; 3) leaflet perforation repair with bovine pericardial patch; and 4) annuloplasty ring implantation.

**Potential Pitfalls:**

Patch repair can fail if the patch is undersized, poorly positioned, or not well integrated, leading to residual regurgitation or early breakdown. Anterior leaflet repairs also carry a risk of systolic anterior motion, and prior surgery may complicate access owing to adhesions.

**Take-Home Messages:**

Mitral valve repair using a pericardial patch is a reasonable option for anterior leaflet perforation, even in complex reoperative settings. Early recognition and a tailored, minimally invasive approach may offer favorable outcomes in selected patients.

## Graphical Abstract

**Figure:**
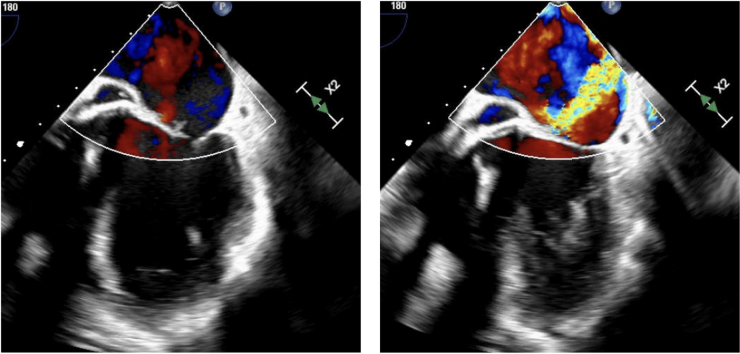

Mitral leaflet perforation is an uncommon but important cause of mitral regurgitation (MR), often linked to infective endocarditis, structural abnormalities, or prior cardiac surgery.^1^^,^^2^ Although valve replacement was traditionally the standard treatment, current evidence favors valve-sparing repair when durable repair is possible (Class I, Level of Evidence: B).^3^^,^^4^ This technique has demonstrated excellent midterm and long-term outcomes, including preserved valve function, low regurgitation recurrence, and low reoperation rates.^5^^,^^6^

**Take-Home Messages**

• Mitral valve repair using a pericardial patch is a reasonable option for anterior leaflet perforation, even in complex reoperative settings.
• Early recognition and a tailored, minimally invasive approach may offer favorable outcomes in selected patients.

In this report, we describe a patient with a complex surgical history, including prior aortic valve replacement and mitral debridement for infective endocarditis, who was found to have anterior mitral leaflet perforation and underwent successful repair through a minimally invasive, valve-sparing approach. This case offers insight into the feasibility and benefits of mitral valve preservation in complex reoperative settings.

## Case Summary

We present the case of a 52-year-old man, an active smoker with a medical history of hypertension, hyperlipidemia, chronic kidney disease, cerebrovascular accident, and depression, who works as a mechanic. Four years earlier, the patient had undergone aortic valve replacement with a bioprosthetic valve, a maze procedure, and mitral vegetation removal during surgery for infective endocarditis at an outside hospital.

**Figure 1:**
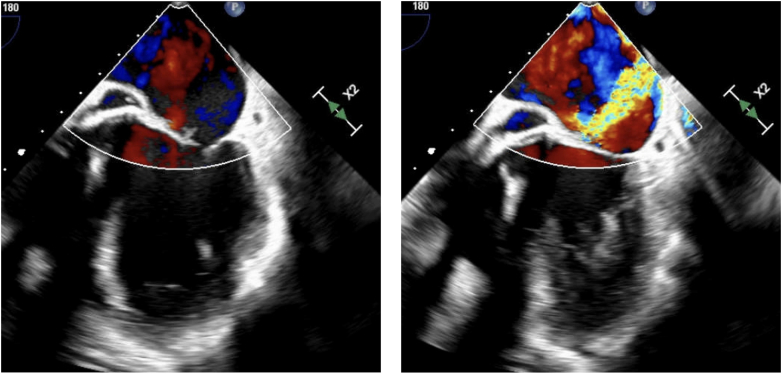
Sequential Echocardiographic Images Demonstrating Anterior Mitral Perforation Associated With Severe Mitral Regurgitation Before Repair

During routine follow-up, he was found to have recurrent mitral insufficiency and was referred to our center. Transthoracic echocardiography revealed severe MR due to a perforation of the anterior mitral leaflet (A2), with otherwise acceptable coaptation and normal prosthetic aortic valve function (Figure 1).

Given the patient's age, occupation, and desire to avoid lifelong anticoagulation, a valve-sparing strategy was pursued.

## Procedural Steps

The procedure was performed under general anesthesia using central aortic cannulation. A narrated overview of the case, including preoperative imaging and intraoperative findings, is available in Video 1. A 5- to 6-cm right minithoracotomy incision was made in the fourth intercostal space, and the pericardium was carefully opened. Dense adhesions from prior surgery were encountered immediately, particularly involving the right lung.

Meticulous dissection was required to free the lung from the chest wall and pericardium. Several visceral pleural perforations were noted, requiring wedge resection of the anterior lung edge using a stapler. The staple line was reinforced with pericardial strips to minimize the risk of postoperative air leaks. Once this was completed, we initiated cardiopulmonary bypass and cooled the patient to 28 °C. The pericardium was further dissected away from the pulmonary veins, a vent was placed through the right superior pulmonary vein, and the aorta was mobilized and cross-clamped.

**Figure 2:**
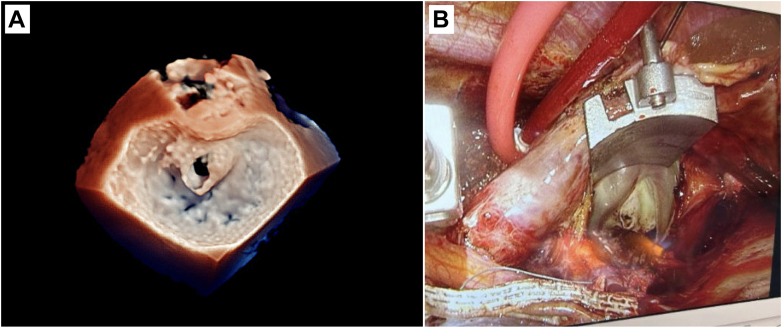
Anterior Mitral Valve Leaflet Perforation Before Repair (A) Three-dimensional echocardiography and (B) intraoperative photograph of the anterior mitral valve leaflet perforation before repair.

The left atrium was accessed through a standard Sondergaard's groove approach. The atrium was elevated with an atrial lift retractor to expose the mitral valve. Inspection revealed a perforation in the anterior leaflet (A2) (Figure 2), with minimal surrounding fibrosis and otherwise healthy leaflet tissue.

**Figure 3:**
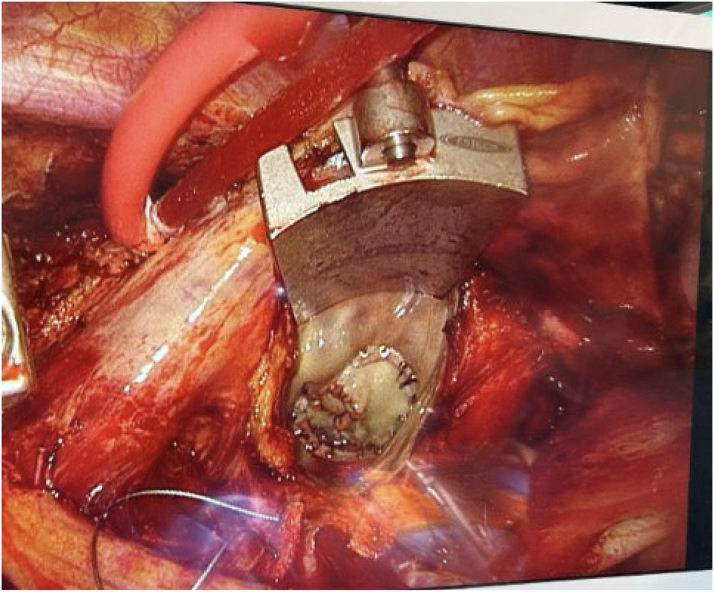
Intraoperative Photograph Showing the Mitral Valve After Successful Repair Using a Pericardial Patch

The perforation was repaired using a 2 × 2 cm bovine pericardial patch sewn with running 4-0 Prolene. Two Tevdek sutures were placed in the annulus and passed through a 34-mm flexible annuloplasty ring, which was secured with the Cor-Knot system (LSI Solutions) (Figure 3).

Intraoperative transesophageal echocardiography confirmed a competent mitral valve with no residual regurgitation, no systolic anterior motion, and normal left ventricular function. The aortic prosthesis remained well seated and functional. The patient was weaned from cardiopulmonary bypass without difficulty. Total cardiopulmonary bypass time was 181 minutes, and aortic cross-clamp time was 101 minutes.

The patient tolerated the procedure well and was transferred to the intensive care unit in stable condition; he was subsequently extubated without complications. He was discharged home on postoperative day 5. At his 1-month follow-up visit, he was recovering well.

## Potential Pitfalls

This case highlights the feasibility and value of a valve-sparing strategy even in a patient with prior aortic valve surgery and mitral debridement. As detailed by El Sabbagh et al,^7^ early recognition and repair of MR, before the onset of irreversible ventricular dysfunction, are crucial to optimize outcomes. Their review supports early surgical intervention in severe primary MR, especially when the likelihood of durable repair is high, as it reduces mortality and improves long-term quality of life.^7^ In our case, timely diagnosis using echocardiography allowed for prompt surgical intervention before ventricular remodeling occurred.

Although minimally invasive valve-sparing techniques offer clear advantages and have demonstrated durable outcomes, they come with important challenges. Patch repairs using autologous pericardium can fail if the patch is too small or poorly positioned, leading to residual regurgitation or early breakdown.^6^^,^^8^ There is also a risk of systolic anterior motion, particularly in anterior leaflet repairs, where altered leaflet geometry can obstruct the outflow tract.^9^ Proper patch sizing and annuloplasty are critical to mitigate these issues. Annuloplasty plays a central role in this context by stabilizing the annular geometry, enhancing leaflet coaptation, and reducing the mechanical stress on the reconstructed tissue. Importantly, it helps to prevent systolic anterior motion by shifting the coaptation point posteriorly and preserving valve competence despite leaflet augmentation. These biomechanical benefits likely contributed to the avoidance of adverse outcomes in our case, even under technically challenging conditions.^10^^,^^11^

In a reoperative setting, a right minithoracotomy avoids the high-risk sternal re-entry. This approach generally provides direct access to the mitral valve without the need for extensive cardiac mobilization;^12^ however, in complex cases, prior surgery can make exposure more challenging. Despite potential challenges, evidence consistently demonstrates favorable outcomes with this approach. A 2018 meta-analysis demonstrated reduced mortality rates (OR: 0.41, 95% CI: 0.18-0.96; *P* = 0.04), shorter hospital stays, and fewer reoperations for bleeding when using a minithoracotomy approach compared with standard sternotomy in a redo setting.^13^ These findings are supported by the retrospective study of Monsefi et al^14^ that included 20 patients who underwent reoperative mitral valve surgery through a right-sided minithoracotomy, with 5% mortality, no neurologic complications, and excellent midterm outcomes. More recently, Zwischenberger et al^15^ in 2024 reported that the 10-year survival rate was significantly higher in reoperative patients undergoing thoracotomy compared with sternotomy (81% ± 3% vs 60% ± 5%, respectively; *P* = 0.006).

In our case, significant lung adhesions from prior surgery made access more challenging and required wedge resection, highlighting how, in complex settings, reoperative minimally invasive procedures may require additional steps to achieve adequate exposure. Nevertheless, the procedure was completed successfully and without complications. This case adds to the growing evidence that mitral valve preservation, when technically feasible, offers advantages even in complex surgical scenarios.

## Conclusions

In relatively young, active patients, valve preservation significantly affects their functional recovery and long-term quality of life. This case report supports the use of minimally invasive, valve-sparing techniques in selected patients.

## Funding Support and Author Disclosures

The authors have reported that they have no relationships relevant to the contents of this paper to disclose.

## Appendix

Video 1 Video Describing the Case Presentation and Surgical Management
